# Terminal ilial intussusception in an adult due to endometriosis

**DOI:** 10.1186/s13104-016-2029-z

**Published:** 2016-04-26

**Authors:** R. K. M. D. C. D. Ranaweera, S. M. K. Gamage, D. H. B. Ubayawansa, M. M. J. Kumara

**Affiliations:** Professorial Surgical unit, Teaching Hospital Galle, Galle, Sri Lanka; Department of Anatomy, Faculty of Medicine, University of Peradeniya, Peradeniya, Sri Lanka

**Keywords:** Adult, Intussusception, Endometriosis, Intestinal obstruction

## Abstract

**Background:**

Intussusception is invagination of a proximal segment of bowel into the distal segment in telescopic manner. Although intussusception is common among children, intussusception secondary to terminal ileal endometriosis in an adult is a very rare encounter. We present such a case of intussusception in a Sri Lankan female.

**Case presentation:**

A 43 year old Sri Lankan female presented to the surgical casualty unit with features of a subacute intestinal obstruction. Her past surgical and medical histories were unremarkable. On examination she was haemodynamically stable with distended abdomen and there was generalized tenderness. There was no guarding or rigidity. No masses were palpable. Bowel sounds were increased. Her urine was negative for Human Chorionic Gonadotrophin hormone. Full blood count revealed an increased white blood cell count with predominant number of neutrophils. Plain abdominal X-ray film showed dilated small bowel loops with empty rectum and distal colon. Patient underwent emergency exploratory laparotomy. An annular growth at terminal ileum was noted. Proximal bowel loops were distended. There was no free fluid in the abdomen. Ileo caecal tuberculosis was suspected and right hemicolectomy was performed. Uterus and bilateral ovaries appeared normal. Post surgical recovery was uneventful. The pathologist has noted endometriosis of terminal ileum contributing to the stricture formation and intussusception at the site. Following recovery patient was referred to a Gynaecologist for management of endometriosis.

**Conclusion:**

Though terminal ileal endometriosis is a very rare cause of intussusception it is important to consider the possibility of it, especially when a female patient of reproductive age presents with symptoms and signs of intestinal obstruction.

## Background

Intussusception is invagination of a proximal segment of bowel into the distal segment in telescopic manner causing intestinal obstruction [1]. This condition is usually seen in children of 6 months to 2 years age group and is rare in adults [1, 2]. Endometriosis is the presence of normal endometrial tissue outside the uterus. Although endometriosis is a common condition among females of reproductive age, intestinal endometriosis is rare [2]. There are several reported cases of endometriosis of sigmoid colon and rectum but cases of small bowel endometriosis are less reported [3, 4]. Intussusception and obstruction due to endometriosis of small bowel is a very rare encounter [1–3]. We present a case of endometriosis of terminal ileum causing terminal ileal intussusception and obstruction at the site. There are only two reported cases of terminal ileal endometriosis causing intussusception of bowel. Therefore this case will be an important addition to the available very short list of relevant literature. Objective of reporting this case is to add another extremely rare case of clinical importance to literature, thereby bringing awareness of such rare presentations and possible misleading points.

## Case presentation

A 43 year old, single and nulliparous female from southern province of Sri Lanka, presented to the surgical casualty ward with generalized colicky abdominal pain and constipation for 7 days duration. However she has passed flatus. She has had nausea and vomiting for 4 days duration which had been exacerbated on the day of presentation. Her usual menstrual periods had a cycle duration of 35–42 days and moderate bleeding lasting for 6–7 days with tolerable lower abdominal and back pain lasting for 3 days from the onset of bleeding. Her last menstrual bleeding had occurred 3 weeks prior. Her past medical and surgical histories were unremarkable. There was no family history of bowel carcinoma or inflammatory bowel pathologies.

On examination patient was afebrile and not pale. She was moderately dehydrated and was in pain. Pulse rate was 130 beats per minute and blood pressure was 100/70 mmHg. Abdomen was distended and there was generalized tenderness. There was no guarding or rigidity. No masses were palpable. Bowel sounds were increased.

Her urine HCG (Human Chorionic Gonadotrophin hormone) was negative. Full blood count revealed increased white blood cell count of 14 × 10^9^/L (per litre) with 80 % neutrophils. Plain abdominal X-ray film showed dilated small bowel loops with empty rectum and distal colon.

A nasogastric tube was inserted and intravenous fluid resuscitation was begun. Exploratory laparotomy was planned. Midline abdominal incision was made and peritoneal cavity was opened into. An annular growth at terminal ileum was noted. Proximal bowel loops were distended. There was no free fluid in the abdomen. Ileo caecal tuberculosis was suspected and right hemicolectomy was performed. Uterus and bilateral ovaries appeared normal.

Post surgical recovery was uneventful. Patient was started on prophylactic antibiotics. Bowel sounds appeared on post operative day 1 and she passed flatus and faeces on post op day 2 and 3 respectively. Patient was discharged from hospital 4 days following surgery and she had no complains on discharge.

The pathologists report explained that on macroscopic examination the terminal ileum had been oedematous and enlarged with a diameter of 4 cm. A stricture had been identified in terminal ileum 2 cm away from caecum. An intussusception had been visualized at the site of stricture. Microscopic examination revealed endometriosis of terminal ileum contributing to the stricture formation and intussusception at the site.

The patient was referred to a Gynaecologist for management of endometriosis.

## Discussion

Small bowel obstruction is a common cause for surgical casualty admissions. It accounts for over 20 % of all hospital admissions due to acute abdominal pain. Malignant growths, strangulated herniae and adhesions are the common causes for small bowel obstruction in adults [5]. Endometriosis is the presence of endometrial tissue outside the uterus. Although endometriosis in pelvis, rectum and sigmoid colon are reported frequently small bowel endometriosis is rarely reported. Occurence of small bowel endometriosis is reported to be only 0.5 % even among diagnosed endometriosis patients. Only 0.15 % of patients with small bowel endometriosis develop small bowel obstruction [2].

Intussusception is a common cause of bowel obstruction in children. Over 95 % of all reported intussusception cases have occured in children, whereas only 1–5 % were reported in adults [5]. In children intussusception is usually idiopathic or secondary to viral infections [6]. However in adults, intussusception is usually due to ‘leading point’ [5, 8, 9]. Leading point usually is a lesion in the lumen of the bowel which interferes with the peristalsis process. Following the interference, bowel segment above the lesion becomes constricted and segment below becomes relaxed. Continuous peristalsis leads to telescoping of the proximal segment (intussusceptum) of bowel into the distal segment (intussuscepiens) resulting in an intussusception [5]. The most common ‘leading points’ in adults are known to be malignant or benign tumors [6]. Bowel tuberculosis is a common differential diagnosis especially in the South Asian region, misleading the surgeons as in this case. In this case terminal ileal stricture caused by the chronic endometriosis has acted as the leading point.

Complications of intestinal endometriosis include intestinal obstruction, hemorrhagic ascites, perforation and intussusception. If not treated immediately intussusception due to endometriosis may lead to intestinal obstruction and gangrene due to impediment of venous followed by arterial blood flow [7]. The typical symptoms suggestive of endometriosis are infertility, dysmenorrhoea and dyspareunia. It is not possible conclude about this patients fertility and sexual intercourse since she is single. In addition, in spite of having longer menstrual cycle duration, patient does not have considerable pain on menstruation. Therefore it is rather difficult to have prior diagnosis of intestinal endometriosis especially in a case like this, as the patient has had no considerably suspicious features suggestive of endometriosis in her menstrual and reproductive histories. However, it is important to consider the possibility of intestinal endometriosis, especially when a female of reproductive age presents with features of intestinal obstruction. This may be of immense value in arriving at a tentative diagnosis.

## Conclusions

Though terminal ileal endometriosis is a very rare cause of intussusception it is important to consider the possibility of it, especially when a female patient of reproductive age presents with symptoms and signs of intestinal obstruction.

## Abbreviations

mmHg: millimetres mercury
HCG: human chorionic gonadotrophin
/L: per litre
cm: centimetres
